# Terbium Ion Doping in Ca_3_Co_4_O_9_: A Step towards High-Performance Thermoelectric Materials

**DOI:** 10.1038/srep44621

**Published:** 2017-03-20

**Authors:** Shrikant Saini, Haritha Sree Yaddanapudi, Kun Tian, Yinong Yin, David Magginetti, Ashutosh Tiwari

**Affiliations:** 1 Nanostructured Materials Research Laboratory, Department of Materials Science and Engineering, University of Utah, Salt Lake City, Utah, 84112, USA

## Abstract

The potential of thermoelectric materials to generate electricity from the waste heat can play a key role in achieving a global sustainable energy future. In order to proceed in this direction, it is essential to have thermoelectric materials that are environmentally friendly and exhibit high figure of merit, ZT. Oxide thermoelectric materials are considered ideal for such applications. High thermoelectric performance has been reported in single crystals of Ca_3_Co_4_O_9_. However, for large scale applications single crystals are not suitable and it is essential to develop high-performance polycrystalline thermoelectric materials. In polycrystalline form, Ca_3_Co_4_O_9_ is known to exhibit much weaker thermoelectric response than in single crystal form. Here, we report the observation of enhanced thermoelectric response in polycrystalline Ca_3_Co_4_O_9_ on doping Tb ions in the material. Polycrystalline Ca_3−x_Tb_x_Co_4_O_9_ (x = 0.0–0.7) samples were prepared by a solid-state reaction technique. Samples were thoroughly characterized using several state of the art techniques including XRD, TEM, SEM and XPS. Temperature dependent Seebeck coefficient, electrical resistivity and thermal conductivity measurements were performed. A record ZT of 0.74 at 800 K was observed for Tb doped Ca_3_Co_4_O_9_ which is the highest value observed till date in any polycrystalline sample of this system.

Thermoelectric (TE) power generation is considered as one of the most promising and emerging clean energy technologies for harvesting electricity from heat without producing any direct emission of greenhouse gases. Absence of moving parts makes TE devices attractive for many applications including automobiles. The thermal-to-electrical conversion efficiency of a TE material is quantified by a dimensionless quantity called figure of merit ZT which at a given temperature T is related to its Seebeck coefficient (S), electrical conductivity (σ), and thermal conductivity (κ) by the relation ZT = S^2^Tσ/κ. One of the major challenges faced today by the thermoelectric community is to practically find stable TE material whose figure of merit is close to 1^1^. Although there are materials whose figure of merit is close to 1, yet their applications are limited due to issues related to their chemical stability and toxicity^2^. At high temperatures these materials degrade/evaporate limiting their application to low working temperatures. Therefore, there is a need to develop TE materials which have high figure of merit and at the same time are chemically stable and non-toxic.

In order to obtain a high figure of merit, the TE material should possess not only a high Seebeck coefficient but also high electrical conductivity and low thermal conductivity. A high electrical conductivity is needed to reduce the joule heating, and a low thermal conductivity is required to maintain a high temperature difference between the hot and cold ends of the TE device. These are the contradictory requirements and there are very few materials which satisfy the above conditions. Layered cobalt oxides are one of such materials. Crystal structure of these materials consists of two alternate CoO_2_ layers sandwiching a block layer comprising of insulating rock salt structure^3^. The first widely studied member of the above family, Na_2_Co_2_O_4_, was reported by Teraski *et al*. in 1997 wherein they demonstrated that this material exhibits a high Seebeck coefficient of 100 μV K^−1^ with a low electrical resistivity of 200 μΩ cm at 300 K^4^. However, sodium being hygroscopic in nature and volatile above 800 °C, limits the use of Na_2_Co_2_O_4_ for practical applications. Therefore, much research has lately focused on the development of other members of layered cobalt oxide family^5^. In 2003, Shikano *et. al.* demonstrated a high figure of merit of ~0.87 at 973 K in single crystal Ca_3_Co_4_O_9_ (CCO)^6^. However, due to their small sizes, single crystal Ca_3_Co_4_O_9_ have limitations for practical applications. Over the last one and half decades or so, tremendous efforts have been put to realize high ZT values in polycrystalline Ca_3_Co_4_O_9_ samples. The enhancement in the thermoelectric performance of polycrystalline Ca_3_Co_4_O_9_ over the years has been summarized in Fig. 1(a)^7^,^8^,^9^,^10^,^11^,^12^,^13^,^14^,^15^,^16^,^17^,^18^,^19^.

Most of the research aimed on enhancing the ZT of polycrystalline Ca_3_Co_4_O_9_, as of yet, has been based on doping approach^5^,^20^,^21^,^22^,^23^,^24^,^25^,^26^,^27^,^28^,^29^,^30^,^31^. The main challenge in this approach is to find the right dopants which increase sample’s ‘S’ and ‘σ’ while at the same time reduce its ‘κ’. So far the partial substitution of Ca^2+^ by heavy trivalent ions, especially rare-earth (RE) ions, has proved to be most effective in enhancing ZT values. The above substitution results in a decrease in the concentration of holes in the material which in turn increases the value of ‘S’. Heavy RE ions also cause scattering of phonons and hence the thermal conductivity of the material goes down. A brief survey of the ZT with the doping of different rare earth elements is shown in Fig. 1(b)^5^,^20^,^21^,^22^,^23^,^24^,^25^,^26^,^27^,^28^,^29^,^30^,^31^.

In this paper, we are reporting the highest value of ZT observed so far for any rare earth doped polycrystalline Ca_3_Co_4_O_9_ sample. Specifically we are showing that when Ca in Ca_3_Co_4_O_9_ is replaced by Tb ions, its ZT value enhances significantly. Furthermore, we have shown that since the ionic size of Tb ions is quite close to that of Ca, a much higher level of dopants compared to other RE ions can be introduced in the lattice before causing the precipitation of the secondary phases. Tb doped Ca_3_Co_4_O_9_ showed a record ZT of 0.74 at 800 K which is the highest value observed till date in this system. Furthermore, compared to other RE elements, Tb is abundant in nature, economical and less toxic, which makes it a potential candidate for practical applications^32^.

## Methods

### Sample Preparation

Polycrystalline ceramic samples of Ca_3−x_Tb_x_Co_4_O_9_ with x = 0–0.7 were prepared by solid state reaction technique. Stoichiometric amounts of Calcium oxide (CaO, Alfa Aesar, 99.9%), Cobalt oxide (Co_3_O_4_, Alfa Aesar, 99.7%) and Terbium oxide (Tb_4_O_7_, Alfa Aesar, 99.9%) were mixed together to obtain a homogenous powder. The resulting powder was then calcined twice at 973 K for 20 hours with intermediate grinding steps. The powder was then compacted into 0.5 inch diameter pellets under a pressure of 45 MPa using a uniaxial press. The compacted pellets were further compressed iso-statically under a pressure of 200 MPa to obtain high and uniform density pellets. Later, the pellets were sintered at a temperature of 1173 K for 20 hours, with re-pelletizing and sintering procedures repeated twice. Finally, the sintered pellets were shaped into rectangular shaped samples of dimensions 10 mm × 4 mm × 2 mm to perform various characterization experiments.

### Characterizations

X-Ray diffraction (XRD) experiments were performed using Philips X’Pert diffractometer with Cu K_α_ radiation. Micro-structural characterization was performed using an FEI Quanta 600 FEG scanning electron microscope (SEM). The SEM images obtained were used to determine the grains and their distribution. The transmission electron microscopy (TEM) including selected area electron diffraction pattern (SAED) and energy dispersive spectroscopy (EDS) was performed using a high-resolution JEOL 2800 S/TEM. The TEM specimens were prepared by ultra-sonication of the crushed powder in ethanol followed by drop casting on a Cu holey carbon grid. X-ray photoelectron spectroscopy (XPS) was performed using a Kratos Axis Ultra DLD system with a monochromatic Al K_α_ source (1486 eV). The component peaks were fitted using Casa XPS software to quantify the elemental composition of the samples. The electrical conductivity, Seebeck coefficient and thermal conductivity were measured over the temperature range 300 K to 800 K. For electrical conductivity measurements, 4-probe technique was employed in which a constant current was passed through the sample using a current source (KI 6220) and the resulting voltage was measured by a nano-voltmeter (KI 2182 A). For Seebeck coefficient measurements, a temperature difference was established between the two ends of the sample and resulting voltage was measured using high-purity platinum wire. Thermal diffusivity was measured by laser flash technique using a pulsed excimer laser (Compex Pro). The heat capacity was measured using a Netzch DSC 3500 Sirus differential scanning calorimeter. By using experimentally measured thermal diffusivity, specific heat and density, thermal conductivity of the samples was determined. The statistical analysis of the measurement result shows uncertainty about 1–2% for electrical conductivity, 2–5% for Seebeck coefficient and thermal conductivity, which is overall about 5–12% for power factor and 7–17% for figure of merit ZT.

## Results and Discussion

### Structural Characterization

**Figure 2:**
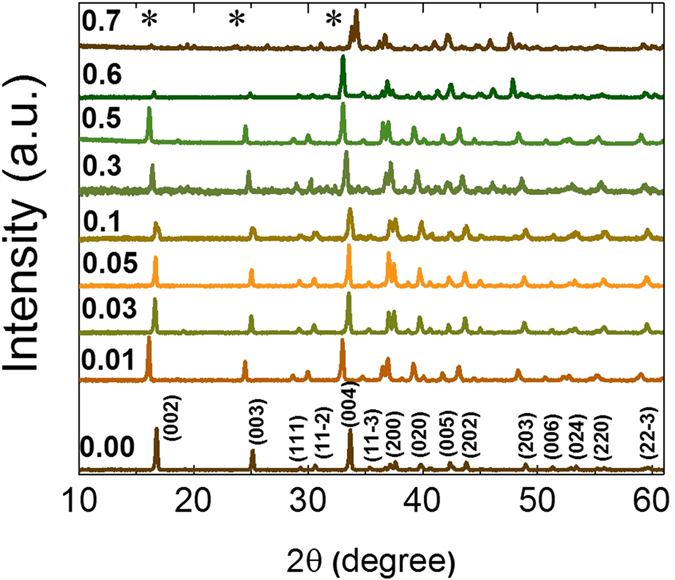
XRD pattern of various Tb doped Ca_3_Co_4_O_9_ samples. *c*-plane oriented grains are dominating in the samples. The asterisks on x = 0.7 show the complete suppression of (00l) peaks and the appearance of additional impurity peaks.

The XRD patterns of the Ca_3−x_Tb_x_Co_4_O_9_ samples are shown in Fig. 2. The data has been plotted with an offset in y-axis for a better visibility. The XRD pattern of the undoped Ca_3_Co_4_O_9_ sample shows all the characteristic peaks as reported in JCPDS database and previous reports^33^,^34^. The pattern remains unchanged till x = 0.5 except for a slight shift in the peak positions towards the higher angle side. This shift indicates a slight decrease in the lattice parameters, which is consistent with the slightly smaller size of Tb ions compared to Ca^2+^ ion. On increasing the Tb content further, for x = 0.6 sample the intensity of the (002) and (003) peaks diminishes un-proportionally while for the x = 0.7 sample, few additional impurity peaks were detected (as marked in Fig. 2). These results indicate that x = 0.5 is the maximum value of Tb content for which sample remains single phase.

**Figure 3:**
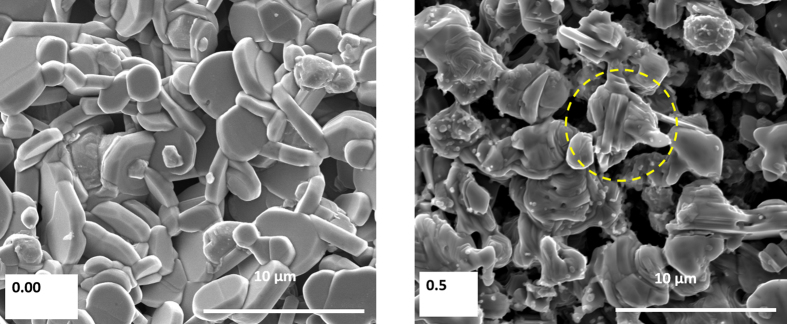
SEM images of: (**a**) Ca_3_Co_4_O_9_ and **(b)** Ca_2.5_Tb_0.5_Co_4_O_9_ samples.

SEM micrographs of x = 0 and 0.5 samples are shown in Fig. 3 (SEM micrographs of the remaining compositions are shown in the Supplementary section). The x = 0 sample showed micron-sized disk like grains which maintained their individuality without much connectivity between the grains. On the other hand, the grains of Tb-doped samples showed slightly fused structure with better connectivity between the grains. All the samples investigated in this study showed relatively low density which was about 70% of the theoretical density^33^.

**Figure 4:**
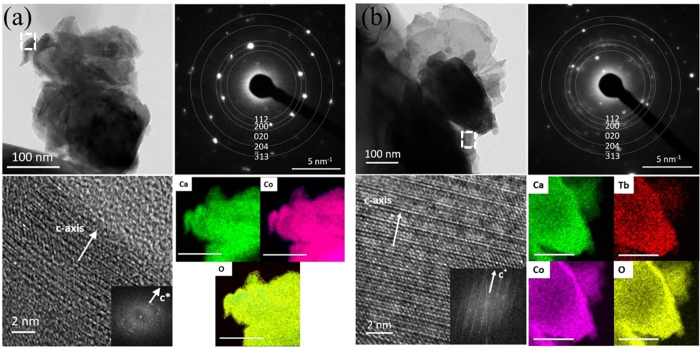
TEM data for: (**a**) Ca_3_Co_4_O_9_ and (**b**) Ca_2.5_Tb_0.5_Co_4_O_9_. In both the cases, the low magnification TEM images are shown in the top-left panels. The HRTEM images (recorded from the small areas marked with broken lines box in low magnification images) are shown in the bottom-left panels. Insets show the FFT patterns of the HRTEM images. Top-right panels show the SAED patterns recorded from the respective samples. Bottom-right panels show the EDS elemental mapping images.

TEM results for x = 0 and 0.5 samples are shown in Fig. 4a and b, respectively. In each case the top-left panel shows low magnification TEM image of the respective samples. The bottom-left panels show the high-resolution TEM (HR-TEM) images recorded from the areas marked with broken lines box in the low magnification TEM images. The insets show the Fast Fourier-transformed (FFT) patterns of the HRTEM images. The layered structure of the material with distinguished CoO_2_ and RS layers can clearly be seen in HRTEM images. The top-right panels show the selected area electron diffraction (SAED) patterns recorded from the respective samples. Since grain sizes in the samples, as seen in SEM micrographs, were of the order of several micrometers, the SAED covered the area containing just few grains. As a result SAED showed speckled ring kind of pattern mixed with diffraction spots of single crystals. The bottom-right panels show the EDS elemental mapping images of Ca, Tb, Co and O in the respective samples. EDS images show the distribution of elements over the whole area examined in the study.

### X-ray photoelectron spectroscopy

**Figure 5:**
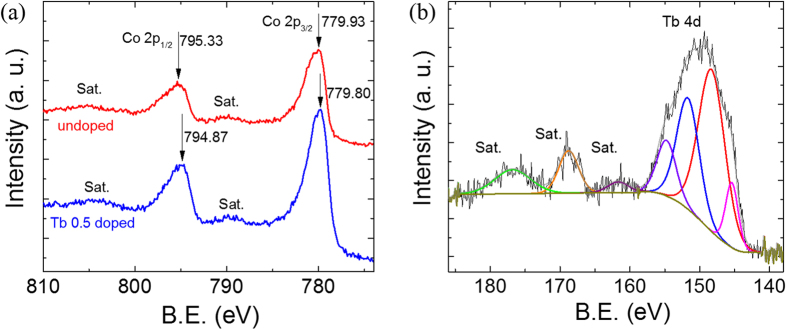
(**a**) High resolution Co 2p_3/2_ and 2p_1/2_ XPS spectra recorded from Ca_3_Co_4_O_9_ and Ca_2.5_Tb_0.5_Co_4_O_9_. (**b**) High resolution Tb 4d XPS spectrum recorded from Ca_2.5_Tb_0.5_Co_4_O_9_.

In order to find the ionic states of the constituent elements, XPS studies were performed. The 2p XPS spectra of Co ions observed from x = 0 and x = 0.5 samples is shown in Fig. 5a. The Co 2p_3/2_ and 2p_1/2_ peaks for the undoped sample lies at 779.93 eV and 795.33 eV, respectively. In literature, it has been reported that for Co^3+^ state, the 2p_3/2_ and 2p_1/2_ peaks occur at 779.6 eV and 794.8 eV, respectively, while for the Co^4+^ state, the 2p_3/2_ and 2p_1/2_ peaks occurs at 781.4 eV and 796.8 eV, respectively^35^,^36^. Since, observed binding energies lye in between the values expected for Co^3+^ and Co^4+^, it is inferred that the Co in the sample exists in the mixed Co^3+^/Co^4+^ valence state. In case of x = 0.5 sample, Co 2p_3/2_ and 2p_1/2_ peaks are observed at 779.80 eV and 794.87 eV, respectively. Though this position is still in between the peaks expected for Co^3+^ and Co^4+^ but is closer to the peak expected for Co^3+^. This indicates that in 0.5 Tb doped sample, the concentration of Co^4+^ ions (and hence of holes) is smaller than that in the undoped sample. In order to understand the observed change in the hole concentration on Tb doping, we recorded Tb 4d XPS spectrum (see Fig. 5b). The observed spectrum comprised of a very broad peak indicating the multiplet splitting in the 4d band. On comparing the XPS data with the corresponding data reported in the literature, it was inferred that the Tb exists in the mixed valence state^37^. The spectral lines in the range 145.4 eV to 148.3 eV arise from Tb^3+^ while those in the range 151.7 eV to 154.8 eV from Tb^4+^. Substitution of Tb^3+^/Tb^4+^ ions in place of Ca^2+^ ions in Ca_3_Co_4_O_9_ is expected to donate electrons which in turn can reduce the net concentration of holes in the system.

### Thermoelectric Characterization

**Figure 6:**
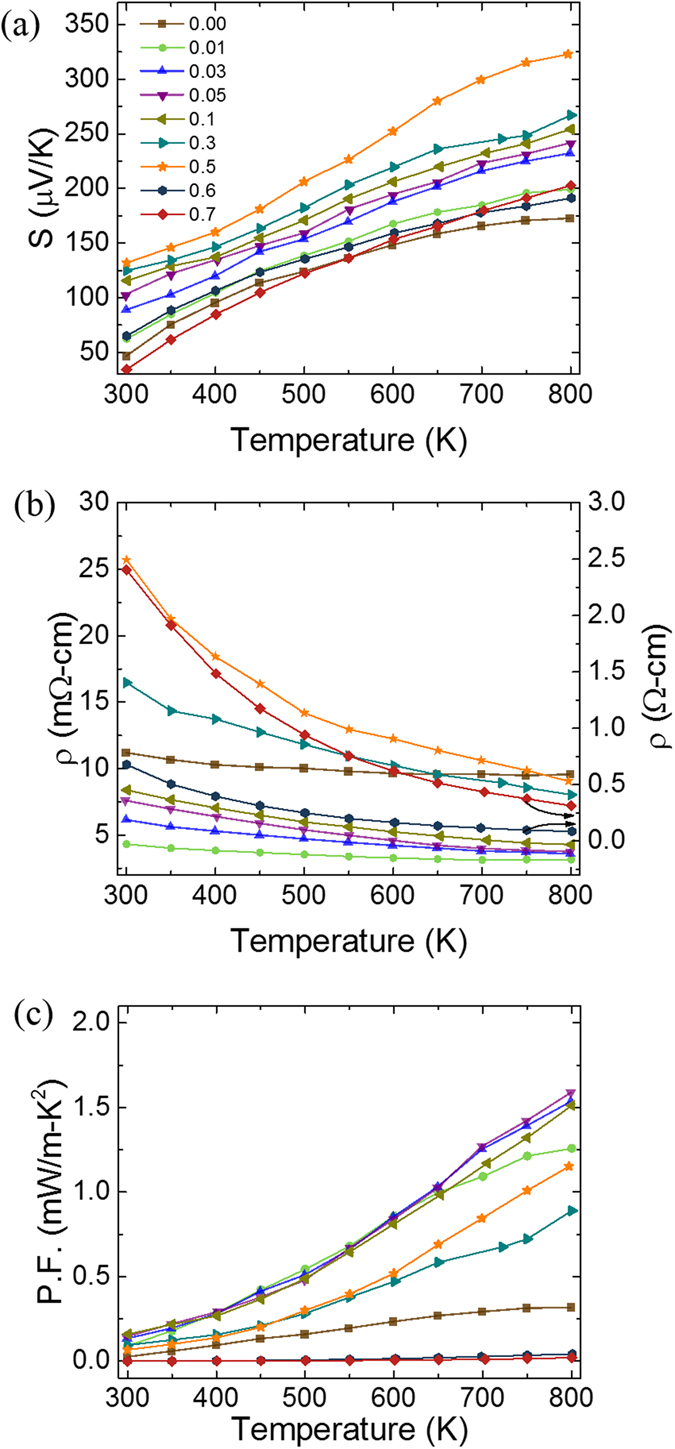
Temperature dependence of: (**a**) Seebeck coefficient; (**b**) Electrical resistivity and (**c**) Power Factor of various Ca_3−x_Tb_x_Co_4_O_9_ samples. Symbols used to represent data points for different samples in (**b**) and (**c**) are the same as in **(a)**. Experimental measurement uncertainties in Seebeck coefficient and electrical resistivity were in the range of 2–5% and 1–2%, respectively.

The temperature dependence of Seebeck coefficient (S) from 300 K to 800 K for the samples is shown in Fig. 6a. As can be seen the sign of the Seebeck coefficient is positive for all the samples indicating that holes are the dominant carriers in the materials. The value of the Seebeck coefficient of the undoped sample at room temperature was 46 μV K^−1^. On introducing Tb in the system, the above value showed a continuous increase till x = 0.5. This increase is consistent with the XPS results, which showed a decrease in the concentration of holes on Tb doping. On further increasing the value of X beyond 0.5, a sudden decrease in the Seebeck coefficient was observed. This decrease is in accordance with the XRD data which showed the formation of impurity phases for x > 0.5. For all the samples, Seebeck coefficient showed a monotonous increase on increasing the temperature. The largest value of Seebeck coefficient ~323 μVK^−1^ was observed for Ca_2.5_Tb_0.5_Co_4_O_9_ at 800 K.

The electrical resistivity vs temperature data for all the samples is shown in Fig. 6b. The overall electrical resistivity decreases with increase in temperature indicating a typical semiconducting-like behavior. Moreover, as the Tb-doping content is increased from x = 0 to 0.5, the electrical resistivity first shows a decrease for x = 0.01 and then increases monotonously. The initial decrease in the electrical resistivity (i.e. increase in electrical conductivity) is in contrast to XPS and Seebeck data, which indicated a reduction in the carrier concentration on introducing Tb in the system. Since electrical conductivity is related to the carrier concentration (n) and the carrier mobility (u) by the relation σ = neu, our results imply that Tb doping enhances the carrier mobility in the resulting system. A similar trend was reported by Sun *et al*. and Bhaskar *et al*. where they observed an increase in the electrical conductivity on doping Bi^3+^ and Dy^3+^ in the Ca_3_Co_4_O_9_^37^,^25^. The increase in electrical resistivity for doping contents x = 0.01 to 0.5, indicates that the decrease in the carrier concentration in this doping range is much more than the increase in the carrier mobility. On further increasing the Tb-doping content beyond x = 0.5, the value of electrical resistivity increases suddenly. Above increase is consistent with the XRD data which showed the formation of impurity phases for x > 0.5. In Fig. 6c, we have shown the variation of power factor (PF = σ.S^2^) with temperature for all the samples. The x = 0.5 sample shows a PF of 1.15 mW m^−1^ K^−2^ is at 800 K. This value is about 4 times higher than the corresponding value for the undoped sample.

### Thermal Conductivity

**Figure 7:**
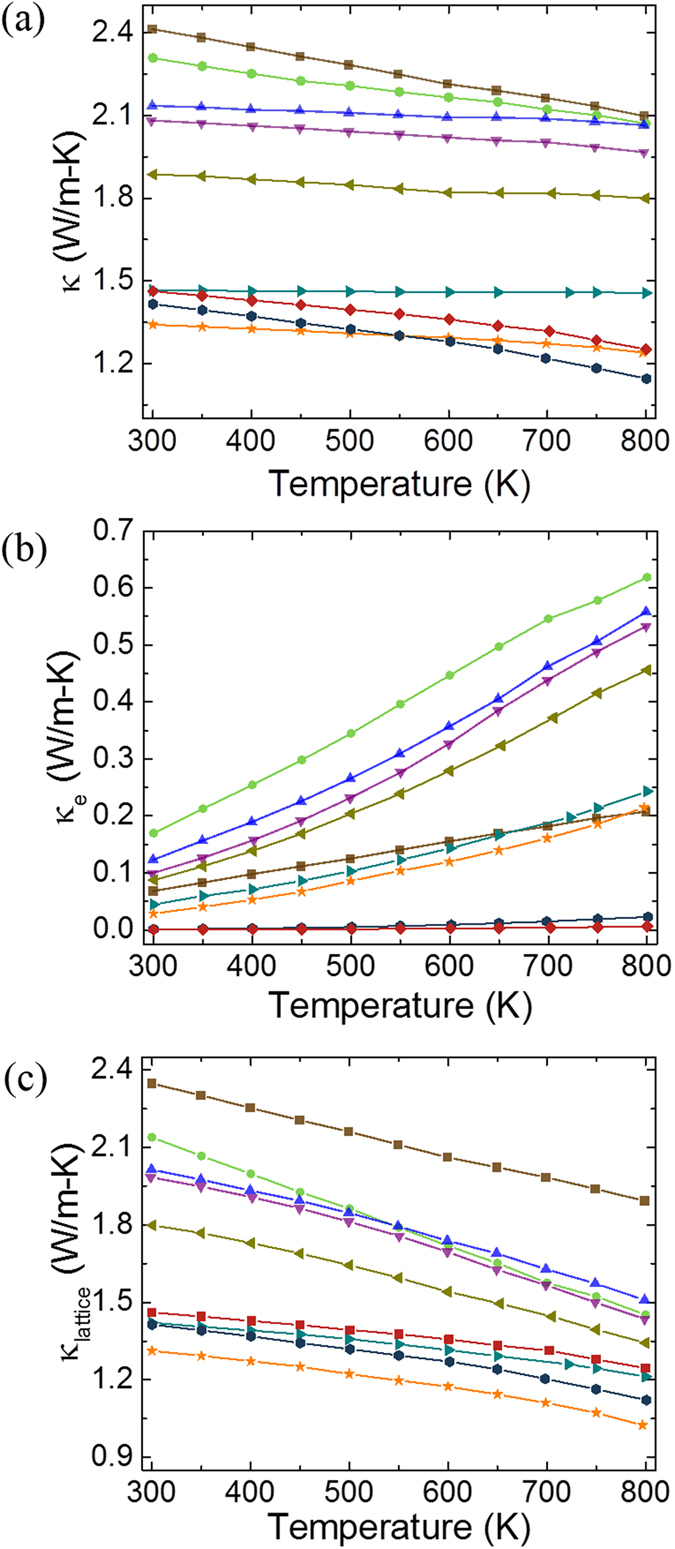
Temperature dependence of: (**a**) total thermal conductivity (κ = κ_e_ + κ_L_); (**b**) electronic part of thermal conductivity (κ_e_); and (**c**) lattice part of thermal conductivity (κ_L_) for various Tb doped Ca_3_Co_4_O_9_ samples. Symbols used to represent data points for different samples are the same as in Fig. 6. Experimental measurement uncertainty in total thermal conductivity was in the range of 2–5%.

Total thermal conductivity (κ) of the samples measured by laser flash technique is shown in Fig. 7a. For the x = 0.0 sample, the value of κ at room temperature (κ_RT_) was 2.41 Wm^−1^K^−1^. On increasing the Tb content, the value of κ_RT_ decreased to 1.34 Wm^−1^K^−1^ for x = 0.5. On further increasing the Tb content, κ_RT_ was found to increase slightly for the x = 0.6 and x = 0.7 samples. For all the samples, the value of κ decreases monotonously with increase in temperature. In order to understand the dominant mechanism responsible for the observed variation in the thermal conductivity, we separated out the electronic (κ_e_) and lattice (κ_L_) components of the thermal conductivities individually. Figure 7b shows the values of κ_e_ determined using the experimentally measured σ values in the Wiedemann-Franz law: *κ*_*e*_ = *L* × *σ* × *T* where, L is the Lorentz number (2.44 × 10^−8^ W.Ω.K^−2^)^38^,^39^. By subtracting the value of κ_e_ from κ, the value of κ_L_ was determined as per the relation: κ_L_ = κ − κ_e_ and is shown in Fig. 7c. As can be seen on increasing the value of x, the value of κ_e_ first increases for x = 0.01 and then starts decreasing while the value of κ_L_ shows a monotonous decrease. However, over the entire temperature range of our study the value of κ_L_ is much higher than that of κ_e_. As a result total thermal conductivity shows a decrease with increase in x.

**Figure 8:**
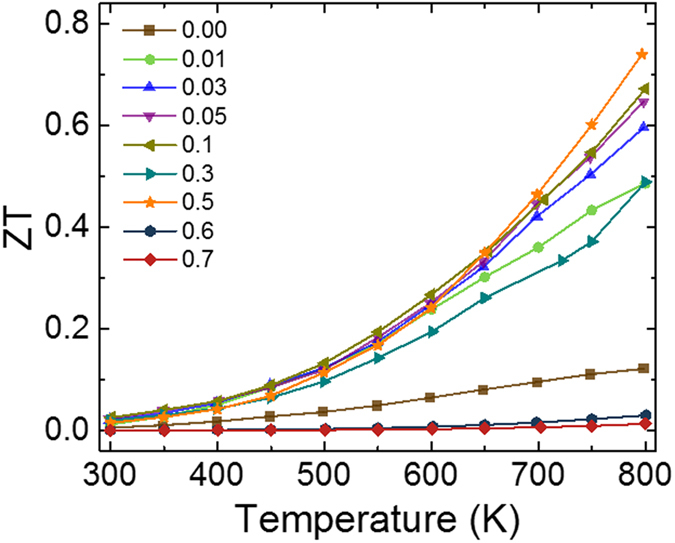
Temperature dependence of ZT for various Tb doped Ca_3_Co_4_O_9_ samples.

Using the experimentally measured ‘S’, ‘σ’ and ‘κ’ values the figure of merit was calculated. The temperature dependence of ZT for all the samples is shown in Fig. 8. The ZT for the undoped sample at room temperature was 2.3 × 10^−3^ which increased to 0.12 at 800 K. On doping Tb in the system, ZT value first increased till x = 0.5 and then started decreasing. For all the samples, ZT showed an increase with increase in temperature. The x = 0.5 sample showed the highest value of ZT attaining a value of 0.74 at 800 K.

## Summary

In summary, we have explored the effect of Tb doping on the thermoelectric response of polycrystalline Ca_3_Co_4_O_9_. A wide range of Tb doping was attempted and state-of-the-art structural characterization and electrical and thermal transport measurements were performed. We found that the maximum concentration of Tb that can be incorporated in the material without causing the precipitation of any impurity phase is x = 0.5. It was observed that Tb doping causes a reduction in the relative concentration of Co^4+^ ions which corresponds to a decrease in the concentration of holes in the material. Reduction in the hole concentration gives rise to a larger ‘S’ while enhanced phonon scattering caused by the heavier Tb ions results in lower ‘κ’. Because of high ‘S’ and low ‘κ’, a high figure of merit of 0.74 at 800 K was observed for the x = 0.5 sample. The enhanced figure of merit at high temperatures along with good chemical stability makes Tb doped Ca_3_Co_4_O_9_ a promising candidate for next generation thermoelectric module applications.

## Additional Information

**How to cite this article:** Saini, S. *et al*. Terbium Ion Doping in Ca_3_Co_4_O_9_: A Step towards High-Performance Thermoelectric Materials. *Sci. Rep.* **7**, 44621; doi: 10.1038/srep44621 (2017).

**Publisher's note:** Springer Nature remains neutral with regard to jurisdictional claims in published maps and institutional affiliations.

## Supplementary Material

Supplementary Information

## Figures and Tables

**Figure 1:**
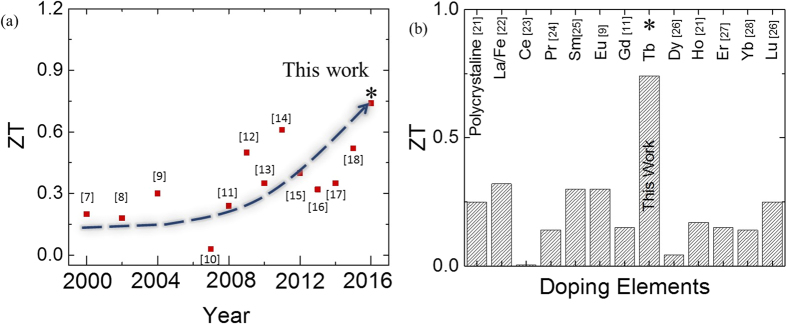
(**a**) Improvement in the ZT of Ca_3_Co_4_O_9_ based thermoelectric materials over the last two decades (refs 7, 8, 9, 10, 11, 12, 13, 14, 15, 16, 17, 18 respectively for each data point); (**b**) A brief survey of the reported ZT values for various rare earth doped Ca_3_Co_4_O_9_ samples. It also shows the ZT value achieved in the present study for Ca_2.5_Tb_0.5_Co_4_O_9_ (marked with asterisk).
