# Supplementary material for: Terbium Ion Doping in Ca3Co4O9: A Step towards High-Performance Thermoelectric Materials
Source: Sci Rep. 2017 Mar 20;7:44621. doi: 10.1038/srep44621 (PMC5357898; doi:10.1038/srep44621)
Supplement: Supplementary Information [file srep44621-s1.doc]

Supporting Information

**Terbium Ion Doping in Ca_3_Co_4_O_9_: A Step towards High-Performance Thermoelectric Materials**

*Shrikant Saini, Haritha Sree Yadanpuddi, Kun Tian, Yinong Yin, David Magginetti, and Ashutosh Tiwari**

Nanostructured Materials Research Laboratory,

Department of Materials Science and Engineering,

University of Utah, Salt Lake City, Utah, 84112, USA


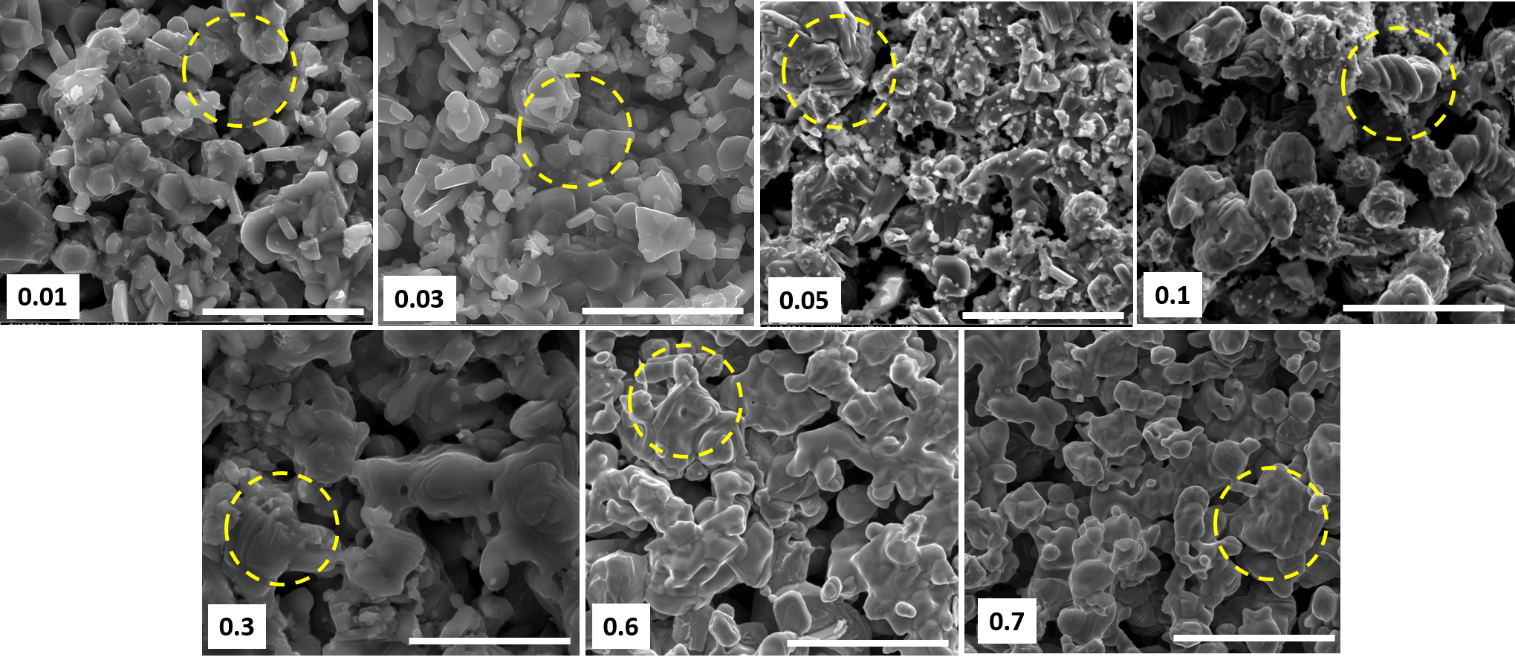


**Figure S1:** SEM images of various Tb doped Ca_3_Co_4_O_9_ samples with the scale bar of 10 µm.
